# Syntactic Priming As a Test of Argument Structure: A Self-paced Reading Experiment

**DOI:** 10.3389/fpsyg.2017.01311

**Published:** 2017-08-15

**Authors:** Isabel Oltra-Massuet, Victoria Sharpe, Kyriaki Neophytou, Alec Marantz

**Affiliations:** ^1^Department of English and German Studies, Rovira i Virgili University Tarragona, Spain; ^2^Neuroscience of Language Lab, NYU Abu Dhabi Research Institute, New York University Abu Dhabi Abu Dhabi, United Arab Emirates; ^3^Serra Húnter Programme, Generalitat de Catalunya Barcelona, Spain; ^4^Neuroscience of Language Lab, Departments of Linguistics and Psychology, New York University New York, NY, United States

**Keywords:** structural priming, comprehension, argument structure, unergativity, transitivity

## Abstract

Using data from a behavioral structural priming experiment, we test two competing theoretical approaches to argument structure, which attribute different configurations to (in)transitive structures. These approaches make different claims about the relationship between unergatives and transitive structures selecting either a DP complement or a small clause complement in structurally unambiguous sentences, thus making different predictions about priming relations between them. Using statistical tools that combine a factorial 6 × 6 within subjects ANOVA, a mixed effects ANCOVA and a linear mixed effects regression model, we report syntactic priming effects in comprehension, which suggest a stronger predictive contribution of a model that supports an interpretive semantics view of syntax, whereby syntactic structures do not necessarily reflect argument/event structure in semantically unambiguous configurations. They also contribute novel experimental evidence that correlate representational complexity with language processing in the mind and brain. Our study further upholds the validity of combining quantitative methods and theoretical approaches to linguistics for advancing our knowledge of syntactic phenomena.

## Introduction

Research has extensively shown that exposure to a syntactic structure influences to different degrees the way we subsequently process a similar structure in comprehension and production in what has been called *syntactic priming, structural priming, or structural persistence* (e.g., Bock, 1986; Bock and Loebell, 1990; Bock et al., 1992, 2007; Branigan et al., 1995, 2000; Pickering and Branigan, 1998, 1999; Hare and Goldberg, 1999; Pickering et al., 2002, 2013; Loebell and Bock, 2003; Ferreira and Bock, 2006; Thothathiri and Snedeker, 2006, 2008a,b; Carminati et al., 2008; Hartsuiker et al., 2008; Pickering and Ferreira, 2008; Tooley et al., 2009; Tooley and Traxler, 2010; Segaert et al., 2012, 2013; Tooley and Bock, 2014; Traxler et al., 2014; Wittenberg et al., 2014). The main goal of this paper is to use the process of syntactic priming as a behavioral tool to test two competing theoretical approaches to argument structure, namely (i) Hale and Keyser's (1993; 1998; 2002) approach as recently developed in Mateu (2002), Acedo-Matellán (2010), Mateu and Acedo-Matellán (2012), and Acedo-Matellán and Mateu (2013), what we will refer to as the *generative semantics* approach to argument structure, and (ii) Marantz (2005; 2011; 2013), which we will call *interpretive semantics* approach. These two theoretical models illustrate two different views of the syntax-semantics mapping. Whereas Acedo-Matellán and Mateu's model operates with semantically unambiguous structures that directly reflect argument/event structure, Marantz's approach contends that syntax does not necessarily start the derivation with a configuration that transparently represents argument/event structure. The latter thus corresponds to an *interpretive semantics* view of syntax, whereby semantics interprets syntactic structures that do not themselves determine meaning; there might be further semantic readjustments or repair strategies at the interface, similar to those postsyntactic processes found at the morphophonology interface. The former approach, Acedo-Matellán and Mateu's, is conceived as a *generative semantics* view of syntax in the sense that syntax generates syntactic structures that determine semantic interpretation in a strict one-to-one meaning structure mapping^1^.

Since these theories attribute different syntactic configurations to transitive structures like (2–6) and make different claims about the relationship between transitive structures and unergatives like (1), they make different predictions about priming relations between these sentence types.

**Table:** 

**Conditions**			**Segment 1**	**Segment 2**	**Segment 3**	**Segment 4**
			**NP**	**V**	**NP(/PP)**	**PP**
(1)	**C1**.	Unergative	The dog	barked	in a quiet park	at night.
(2)	**C2**.	Cognate	The man	dozed	a restful doze	on the train.
(3)	**C3**.	Creation	The cook	baked	a carrot cake	with spelt flour.
(4)	**C4**.	Location/Locatum	The girl	saddled	a wild horse	in the farm.
(5)	**C5**.	Strong transitives	The athlete	ignored	a slight niggle	in his knee.
(6)	**C6**.	*With*-Small clause	The worker	loaded	a rail wagon	with hay.

In the generative theory, unergatives (1) are analyzed as derived from transitive configurations, as is standardly assumed since Hale and Keyser (1993), and pattern with cognate object constructions (2) as well as with verbs of creation (3), thus predicting syntactic priming among these sentence types but not between these sets and the remaining types (4–6). The latter are assumed to select for a small clause type complement structure, and are therefore predicted to prime among them in this model. On the other hand, the interpretive account does not predict structural priming between the unergatives (1) and the surface transitives, (2–5), nor between complex complement constructions (6) and the other surface transitive sentences. In this model, sentence types (2–5) are analyzed as transitive configurations, whereas (6) would pattern with double object constructions, as suggested and analyzed in Bruening (“Depictive Secondary Predicates, Light Verb Give, and Theories of Double Object Constructions,” unpublished manuscript, University of Delaware), and unergatives in (1) are not generated as underlying transitive configurations. This means that the interpretive approach does predict some cases of priming that the generative model does not; specifically, the former predicts priming between sets (2–3) and (4–5), which are considered to display distinct underlying structures in the latter account.

In order to test these two hypotheses we ran a self-paced reading language comprehension study with 600 subjects over Mechanical Turk. The large number of subjects allows us to model the reading times at the direct object or first PP (Segment 3) and at the second PP (Segment 4) of the same sentences as a function of the structure of the immediately preceding sentence, testing for structural priming within and across sentence types. We conducted a series of statistical analyses and report here the results of two ANCOVAs (Analysis of Covariance) and a linear mixed effects regression analysis on the reading times at Segment 3.

A major headline that can be derived from this study is that we do see syntactic priming effects at all in the context of a behavioral comprehension study on structural priming that uses unmarked unambiguous structures without lexical repetition, i.e., what has been termed lexical boost or lexical enhancement. In addition, our analysis shows a significant effect of the interaction between conditions–the different types of structures as grouped by the different theories–and priming in trials preceded by two trials of the same category in the interpretive model but not in the generative model, which suggests a potentially stronger predictive contribution of the former model over the latter model. More generally, our experimental study supports the validity of quantitative approaches that combine psycholinguistic methodology with sound theoretical hypotheses about the representation and processing of syntactic phenomena for the study of I-language (Chomsky, 1986, p. 21ff).

### Structural priming

The novelty of the self-paced reading syntactic priming effects reported in this study is that we do observe syntactic priming effects at all in a study of structural priming in comprehension with unambiguous active sentences without a lexical boost. Let us first summarize relevant aspects of structural priming as a method to test for syntactic structure to set the context of this study.

Our basic initial observation is that the interpretive and the generative models make different predictions with respect to structural priming, the tendency to more quickly repeat or better process a sentence because of its structural similarity to a previously experienced “prime” sentence. Structural or syntactic priming has been studied across modalities, both in production and comprehension, in behavioral studies. On the one hand, there is consensus that syntactic priming effects in production occur without lexical boost, so that when there is lexical repetition in production, priming effects are boosted or enhanced, e.g., Pickering and Branigan (1998), Segaert et al. (2012), but this is not required to find priming effects. We note here that Pickering and Branigan (1998), in an experiment on completing sentence fragments, report that there is priming without lexical repetition in production only when the target sentence is primed with 2 sentences (but see Mahowald et al., 2016, for a recent meta-analysis that reviews and assesses the current state of knowledge on syntactic priming in language production). On the other hand, most works on syntactic priming in comprehension from different perspectives agree that this is strongly dependent on lexical repetition, e.g., Pickering and Traxler (2004), Branigan et al. (2005), Melinger and Dobel (2005) Arai et al. (2007), Traxler and Tooley (2007, 2008), Tooley et al. (2009), Segaert et al. (2012) and Segaert et al. (2013). That is, exposure to a syntactically related prime sentence leads to a faster reading of a target sentence only if there is lexical overlap of the main verbal head. However, recent studies on structural priming have challenged this view by reporting syntactic priming that is independent from verb repetition in comprehension, specifically Thothathiri and Snedeker (2008a,b), Traxler (2008b), Pickering et al. (2013), and hence also from processing modality, as in Tooley and Bock (2014). We consider here some of the studies on syntactic priming in comprehension in more detail.

Among those that do not observe structural priming in comprehension, Pickering and Traxler (2004) report that there is no priming without lexical boost in this modality on the basis of a reading task with eye tracking recording with sentences containing a reduced relative (cf. Traxler, 2008a). Hence, despite all having the same structure, the sentence in (7a) would prime only (7c), where the main verb is the same, but not (7b).

(7)
  - The man watched by the woman was tall.
  - The child cleaned by the girl was covered in chocolate (TARGET-No lexical boost).
  - The mouse watched by the cat was hiding under the table (TARGET-Lexical boost).

Arai et al. (2007) report results from two experiments where they investigated whether there is priming during comprehension in ditransitive sentences. Using a visual-world paradigm, whereby participants anticipation of linguistic information was monitored through eye-movement, they observed a priming effect similar to that in production, but only when the verb was repeated between prime and target; that is, the priming effect is completely lexically dependent according to these authors.

Although Segaert et al. (2013) report no differential effects across modalities in an fMRI neuronal study of active and passive sentence comprehension and production, they also point out that there is no syntactic priming among active sentences in the absence of lexical boost of the main verbal head word, even though there is priming among passive structures. Although this is not a behavioral study, but an event-related fMRI study investigating syntactic priming and lexical boost effects on the neuronal activity in brain regions processing syntactic structures (left IFG and left MTG), it bears directly on our observation that there are priming effects without lexical boost among basic active sentences, even if only after two previous primes. They measure fMRI adaptation of neural activity to repetition of verb-headed syntactic constructions, and report that “there was fMRI adaptation to syntactic repetition when actives had a repeated verb, but no fMRI adaptation to syntactic repetition when actives had a novel verb.” In the case of passives, “there was fMRI adaptation to syntactic repetition both for passives with a repeated verb and for passives with a novel verb.”

More recently, in an eye tracking identification experiment with children, Thothathiri and Snedeker (2008a) find priming effects without lexical repetition in comprehension. As pointed out in Tooley and Traxler (2010), these effects are found in the context of two primed sentences. However, and perhaps more importantly, these same authors further point out that children's identification involved acting out target sentences with toys, which could potentially be said to invoke some sort of covert production component, in the sense that acting out might involve mechanisms involved in production.

Traxler (2008b) reports the first evidence of between-sentence structural priming in online sentence comprehension without lexical overlap using eye-tracking, where a sentence like (8a), but not sentence (8b), would prime the target sentence (8c), because they both have the same structure, which is different from sentence (8b).

(8)
  - The chemist poured the fluid in the beaker into the flask earlier (PRIME).
  - The chemist poured the fluid into the flask earlier (PRIME).
  - vendor tossed the peanuts in the box into the crowd during the game (TARGET).

However, Traxler himself already points out that given that priming here involves adjunct relations and that previous experiments report the impossibility of structural priming of arguments without lexical boost in comprehension, a difference in syntactic processing of arguments vs. adjuncts may be at stake in this case.

Pickering et al. (2013) observe structural priming in both lexically independent and lexically dependent comprehension in a study based on a sentence-picture matching task with ambiguous PP attachment, which can be either high (modifying the verb) or low (modifying the object), as in (9) below. They show that processing is sensitive to the (lexically specific or lexically independent) frequency of an alternative structural analysis, whether through immediate exposure (immediate priming) or via long-term priming, i.e., after some unrelated intervening sentences (persistence of priming).

(9)
  - The policeman is thumping the soldier with the gun (PRIME–lexically independent).
  - The policeman is prodding the doctor with the gun (PRIME–lexically dependent).
  - The waitress is prodding the clown with the umbrella (TARGET).

Finally, Tooley and Bock (2014) examine structural priming with and without verb repetition in both reading comprehension and spoken production, using the same prime presentation procedure, the same syntactic structures (reduced relatives, RR, and main clauses, MC), the same sentences, and the same group of participants. They report abstract structural priming in both modalities without significant comprehension vs. production differences in terms of lexical dependency. The first four sentences are primes, while the last two are targets.

(10)
  - The speaker selected by the group gave a great talk (RR-same–PRIME).
  - The speaker picked by the group gave a great talk (RR-diff–PRIME).
  - The group selected the speaker who gave a great talk (MC-same–PRIME).
  - The group picked the speaker who gave a great talk (MC-diff–PRIME).
  - The architect selected by the firm had years of experience (RR-TARGET).
  - The firm selected the architect who had years of experience (MC-TARGET).

We note that the kinds of stimuli that have been used in structural priming studies are mostly items that require some process of disambiguation. So, what all works have in common is that they observe–or fail to observe- priming effects following syntactically complex material, what Tooley et al. (2009) call “difficult and ambiguous sentence structure,” sentences that are difficult to process and may need re-parsing because up to a specific point they can receive more than one interpretation. Most research, if not all, on structural priming in sentence comprehension is concerned with how subjects resolve syntactic ambiguities or process complex sentences in incremental sentence processing. These include reduced relatives of the type in (10), which have received the most attention to date in comprehension studies, garden-path sentences, like (11), cases of ambiguous high- or low-PP attachment, as in (9), ambiguous double object vs. dative construction, (12), ambiguous datives vs. locatives, (13), or ambiguous locatives vs. passives, (14).

(11) **Garden-path sentences** (Branigan et al., 1995).
  - While the woman was eating the creamy soup went cold.

(12) **Double Object vs. Dative constructions** (Thothathiri and Snedeker, 2008a,b).
  - Give the bird the dog bone.
  - Give the bird house to the sheep.

(13) **Datives vs. Locatives** (Bock and Loebell, 1990).
  - The wealthy widow drove her Mercedes to the church (PRIME).
  - A rock climber sold some cocaine to an undercover agent (TARGET).

(14) **Locatives vs. Passives** (Bock and Loebell, 1990).
  - The foreigner was loitering by the broken traffic light (PRIME).
  - The referee was punched by one of the fans (TARGET).

The case in (15) is different. Segaert et al. (2012, 2013) observe that whereas passive structures prime passives, active primes do not have any effect, which seems to argue for a higher priming power of marked structures like passive over unmarked active sentences.

(15) **Actives vs. Passives** (Segaert et al., 2012, 2013).
  - The woman serves the man.
  - The man is served by the woman.

Even though ambiguity plays no role in this last case, it confirms, then, that structural priming studies share complexity of processing as a fundamental premise to test their priming hypotheses.

One of the main goals of our experimental study is to show that there is priming in unmarked non-incrementally disambiguating contexts, i.e., in simple active sentences.

### Persistence of priming

Another important feature worth bearing in mind is the persistence of priming, since the design of our experiment is a cumulative running priming paradigm where each target sentence also serves as a prime sentence for the next target sentence. This raises the question of the effects of short-term priming vs. long-term priming. Syntactic priming that persists across unrelated intervening sentences has generally been observed in production (e.g., Bock and Griffin, 2000). All the work we have found on long-term priming in comprehension seems to involve the repetition of the verbal head. On the one hand, Hartsuiker et al. (2008), using a picture description task, show that an enhanced priming effect due to lexical boost does not persist across any number of intervening structures in production. On the other hand, Carminati et al. (2008), using an eye tracking identification task, report that lexically dependent syntactic priming effects persist across two intervening sentences in comprehension. Also in Pickering et al. (2013), it is shown that priming persists with lexical repetition over intervening material in comprehension. More recently, Tooley et al. (2014) have observed structural persistence between prime and target across unrelated filler sentences in sentence priming both in production and comprehension on the basis of event-related potentials (ERP) and eye tracking measures. In their experiments, they use prime sentences containing a reduced relative clause, i.e., a complex and ambiguous structure. We do not consider persistence of priming across intervening sentences in our study, since it is still to be determined whether there is priming at all in comprehension, and whether this persists across intervening material when priming with unmarked unambiguous structures.

### Two theories of argument structure

As pointed out in Marantz (2005) (see also Poeppel and Embick, 2005), generative grammar can and should serve as a source of theoretical hypotheses about the representation of language in the mind and brain and how this is processed, to be formally assessed through standard experimental methods. In this paper we take two competing theories of argument structure, (i) Acedo-Matellán (2010); Mateu and Acedo-Matellán (2012), and Acedo-Matellán and Mateu (2013), and (ii) Marantz (2005; 2011; 2013) and test their claims and predictions with respect to the representation and processing of syntactic argument structure. Both theories are framed within Chomsky's Minimalist Program, and they both adopt a neoconstructionist view of syntax, whereby argument structure is not lexically projected^2^ but created in the syntax by the computational system, a single generative engine for all structure building where minimal units of syntactico-semantic features are combined through the operation of *merge* to create hierarchical syntactic structures that will then receive a semantic and phonological interpretation. Such a basic assumption makes them especially suited for the application of the standard psycholinguistic methodology that correlates representational complexity with computational complexity in the brain, i.e., the hypothesis that “the longer and more complex the linguistic computations necessary to generate the representation—the longer it should take for a subject to perform any task involving the representation” (Marantz, 2005, p. 439). That means that specific differences such as how to merge a root in syntax, whether as a complement or as an adjunct (Acedo-Matellán, 2014), can be reduced to differences in surface syntactic representations of verbal argument structure in the sentences under study^3^. As pointed out in the literature on structural priming, syntactic priming is sensitive or attributable to surface structure, not to abstract structure (e.g., Bock et al., 1992; Pickering et al., 2002; Pickering and Ferreira, 2008; Wittenberg et al., 2014). In this respect, it is worth emphasizing that in both models, the proposed structures are surface structures^4^.

Such fundamental assumptions and similarities between both theories allow us to make use of structural priming as a tool to test a variety of unergative and transitive configurations by measuring reading times at the point where both theories differ in the representation of those syntactic structures, namely between the verb and the first complement (Segment 3).

Before going into the details of our experimental study, the remainder of this section briefly reviews the main claims about the syntax of transitive and intransitive predicates made in the two theoretical models of argument/event structure under study and their predictions with respect to structural priming.

#### The generative approach to argument structure: Hale and Keyser (1993, 1998, 2002); Acedo-Matellán (2010); Mateu and Acedo-Matellán (2012); Acedo-Matellán and Mateu (2013)

In this strict configurational model of argument structure, compositional semantics is directly read off the syntactic structure. Leaving aside unaccusative structures, the configurations advanced in Acedo-Matellán and Mateu's work for the sentence types under study are (16–18).

In the case of unergatives in (16), already since Hale and Keyser's work, the root, √, is generally understood as merged in the complement position of a functional head *v*. The phonological material of the root is then incorporated into this null verbal head *v*. As pointed out in Acedo-Matellán (2010, pp. 53–54), “the structure of unergative verbs as transitives is forced by the properties of the system: it is not possible for a functional head to project a specifier without projecting any complement, since the first DP/root merged with a functional head must be its complement.” This also includes cognate object constructions, which would also have a configuration as in (16b).

(16) Unergative, cognate object, and transitive verbs of creation and consumption.
  - Sue danced.
    [_*v*P_ [_DP_ Sue] [_*v*_' *v* √DANCE]].
  - Sue did a dance.
    [_*v*P_ [_DP_ Sue] [_*v*_' *v* [_DP_ a dance]]].

The syntactic structure in (16), [*v* + DP/√], is thus the configuration attributed to unergatives (C1), cognate object structures (C2) and creation verbs (C3) in this model.

On the other hand, (a)telic transitive events, exemplified in (17–18), are all derived from a small clause predicate configuration-whether simple, with a single PlaceP, or complex, with a Place P c-commanded by a PathP (cf. Jackendoff, 1973; Cinque and Rizzi, 2010). In both cases, there is a Figure that moves with respect to a potential Ground (Talmy, 1975). A single relational functional (prepositional) head *p* (Hale and Keyser's central coincidence P), interpreted as a PlaceP, introduces a Figure-Ground configuration that establishes a location or state. If further c-commanded by a second head *p* (Hale and Keyser's terminal coincidence P), this is interpreted as a PathP and introduces a transition that encodes the change. As with unergatives, the root is merged in complement position of the lower null functional *p* head and the phonological material of the root is then successively merged up to the null verbal head.

(17) Atelic transitive events.
  - Sue pushed the car.
    [_*v*P_ [_DP_ Sue] [_*v*_' *v* [_PlaceP_ [_DP_ the car] [_Place'_ Place √PUSH]]]].
  - Sue lengthened the rope (for 5 min).
    [_*v*P_ [_DP_ Sue] [_*v*_' *v* (= -en) [_PlaceP_ [_DP_ the rope] [_Place'_ Place √LONG]]]].

(18) Transitive events of change of state or location.
  - The strong winds cleared the sky.
    [_*vP*_ [_DP_ The strong winds] [_*v*_' *v* [_PathP_ [_DP_ the sky] [_Path'_ Path [_PlaceP_ [_DP_ the sky] [_Place'_ Place √CLEAR]]]].
  - Sue shelved the books.
    [_*vP*_ [_DP_Sue] [_*v*_' *v* [_PathP_ [_DP_ the books] [_Path'_ Path [_PlaceP_ [_DP_ the books] [_Place'_ Place √SHELF]]]].

Thus, all telic and atelic structures are assigned a syntactic configuration where a null verbal head *v* takes a small clause structure, a *p*P, in complement position, which will be a PlaceP for atelic predicates or a PathP with telic predicates, i.e., a small clause configuration in both cases. This is the structure attributed to location/locatum predicates (C4), like *They saddled the horse*, and strong transitive predicates (C5), like *He ignored the* truth, despite their surface appearance as simple transitive sentences. *With*-small clauses (C6) would also have this syntactic representation, the difference being that the preposition in this case is phonologically realized, not null, and there is therefore no conflation.

#### The interpretive approach to argument structure: Marantz (2005, 2011, 2013)

On the basis of empirical evidence based on the syntax and semantics of *re*-affixation, the interpretation of roots in denominal verbs and restrictions on the interpretation of verbal compounds, Marantz (2011) argues that roots cannot merge as complements of a null functional head, as in Acedo-Matellán and Mateu's structures (16–18), but must merge as event modifiers, i.e., as adjuncts.

We review here the empirical argument based on *re*- affixation. *Re-* prefixation distinguishes between unergative and transitive structures, as in (19), and between verbs selecting a single direct object and those that take two in a small clause configuration, as in (20). On the one hand, restitutive *re-* is restricted to verbs with an underlying direct object (Horn's, 1980; generalization); on the other hand, that direct object must be the sole obligatory constituent within the VP (Wechsler's, 1989 generalization). Hence, the ungrammaticality of (19b) must thus be due to the absence of an underlying object, whereas the grammaticality of (20c) argues against its alleged status as a small clause predicate.

(19)
  - John danced.
  ^*^John re-danced.
  - John re-danced a dance first performed by his distant ancestors.

(20)
  - John put the display ^*^(on the table).
  ^*^John re-put the display on the table.
  - John re-shelved the books.

This means that the root *dance* cannot have been generated in the complement position of the verbal head *v*, because there is no direct object present that *re-* can target in (19a); likewise, *shelve* cannot have a small clause configuration, as proposed in Hale and Keyser and Acedo-Matellán and Mateu, since it does take a direct object that *re-* can target. Marantz concludes that unergatives are plain intransitive predicates, whereas sentence types C2-C5 contain plain transitive predicates, i.e., verbs of creation and incremental themes, unergative verbs with a cognate object, strong transitives, as well as atelic and telic transitives—which includes location and locatum predicates. The structure is illustrated in (22) for a predicate like *hammer the nail* in (21); the root *hammer* modifies the event introduced by *v* in (22), which selects an internal argument DP, *the nail*.

(21) hammer the nail.
(22) 
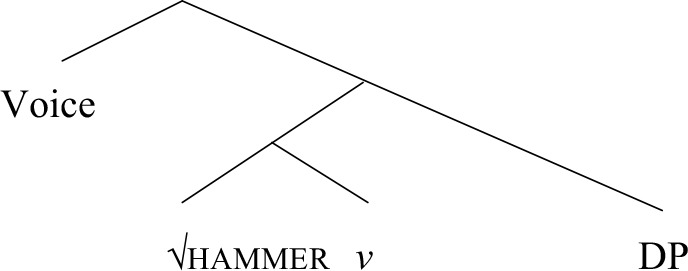

#### Predictions of each model

Since these two theoretical approaches to argument structure attribute different configurations to (in)transitive structures, they make different claims about the relationship between them, and therefore make different predictions about priming relations between these sentence types.

In the *generative* model, unergative verbs (C1) share their transitive syntactic configuration with cognate objects (C2) and verbs of creation (C3), whereas location/locatum structures (C4) and strong transitives (C5) pattern with predicates containing a *with*-small clause (C6). In the *interpretive* model, however, the grouping is organized in three different sets, where cognates (C2), creation verbs (C3), location/locatum (C4) and strong transitives (C5) pattern together in a group separate from unergatives (C1) and small clauses (C6). These differences are represented in Table 1, where we have identified each sentence type as a priming condition, C1–C6.

**Table 1:** Priming conditions, sentence types and groupings by theory.

	**VERB TYPE**	***Generative***	***Interpretive***
C1	UNERGATIVE VERB		
	The dog barked in quiet parks at night.		*v*
C2	COGNATE OBJECT		
	The man dozed a restful doze on the train.	*v* + √/DP	
C3	CREATION		
	He baked a delicious cake with spelt flour.		*v* + √/DP
C4	LOCATION/LOCATUM		
	They saddled a wild horse in the farm.		
C5	STRONG TRANSITIVES		
	He ignored a slight niggle in his knee.	*v* + SC	
C6	*WITH*-SMALL CLAUSE		
	They sprayed a cookie sheet with vegetable oil.		*v* + SC

Given the 6 sentence types we have singled out and the different structural configurations they are assigned in each theory, we identified the divergent individual priming predictions by sentence type made by each model. These are summarized in Table 2. Here we leave aside default identity priming for each individual condition, as well as predictions shared by both models, e.g., priming between C2 and C3. Thus, under structural priming conditions, we would mainly expect faster reading times for the first constituent after the main verb—Segment 3—if the sentence involved follows one (or two) sentences of the same structural type. This is the place where the two models structurally differ with respect to the type of complement, a DP or a small clause. That is priming effects would show up as an effect of the primed/unprimed variable of interest-indicated as checks or crosses on Table 2—based on each theoretical model.

**Table 2:** Priming relations-predictions of each model by individual sentence types.

	**PRIME**	**TARGET**	***Generative***	***Interpretive***
C1>C2	UNERGATIVE	COGNATE	✓	✗
C1>C3	UNERGATIVE	CREATION	✓	✗
C2>C1	COGNATE	UNERGATIVE	✓	✗
C2>C4	COGNATE	LOCATION/LOCATUM	✗	✓
C2>C5	COGNATE	STRONG TRANSITIVE	✗	✓
C3>C1	CREATION	UNERGATIVE	✓	✗
C3>C4	CREATION	LOCATION/LOCATUM	✗	✓
C3>C5	CREATION	STRONG TRANSITIVE	✗	✓
C4>C2	LOCATION/LOCATUM	COGNATE	✗	✓
C4>C3	LOCATION/LOCATUM	CREATION	✗	✓
C4>C6	LOCATION/LOCATUM	*WITH*-SMALL CLAUSE	✓	✗
C5>C2	STRONG TRANSITIVE	COGNATE	✗	✓
C5>C3	STRONG TRANSITIVE	CREATION	✗	✓
C5>C6	STRONG TRANSITIVE	*WITH*-SMALL CLAUSE	✓	✗
C6>C4	*WITH*-SMALL CLAUSE	LOCATION/LOCATUM	✓	✗
C6>C5	*WITH*-SMALL CLAUSE	STRONG TRANSITIVE	✓	✗

When considered in terms of the structural groupings and the predictions of each theory with respect to structural priming effects within and across sentence types, the differences between the two theoretical models are summarized in Table 3. Thus, the *generative* model predicts priming (i) among unergatives, cognate object constructions and creation verbs and (ii) among location/locatum structures, strong transitives, and structures containing a *with*-small clause. However, the *interpretive* theory predicts priming only (i) among cognate object structures, creation verbs, location/locatum predicates and strong transitives, while (ii) unergatives, and (iii) *with*-small clauses would not show priming effects in prime/target interactions with other sentence types.

**Table 3:** Priming relations-predictions of each model by sentence groupings.

	**PRIME/TARGET AMONG THEMSELVES**	***Generative***	***Interpretive***
C1-C2-C3	UNERGATIVE–COGNATE–CREATION	✓	**✗**
C4-C5-C6	LOCATION/LOCATUM–STRONG TRANSITIVES–*WITH* SMALL CLAUSE	✓	**✗**
C2-C3-C4-C5	COGNATE–CREATION–LOCATION/LOCATUM–STRONG TRANSITIVES	**✗**	✓

In the statistical analyses we discuss in the following sections we analyze the priming relations predicted in Table 3, rather than the individual priming relations listed in Table 2.

## Materials and methods

### Participants

We distributed our study via Amazon Mechanical Turk to 600 subjects, from which we obtained 460 full datasets^5^. We restricted this to participants from the U.S and those that had a 95% or greater HIT acceptance rate. Data was processed before starting the analysis, and all non-native English speakers were excluded, together with those that spoke more than one language, English, leaving only 390 monolingual native English participants. Within these 390 datasets, only 375 were unique participants; hence, duplicate participants were excluded as well, and only their first set of data was taken. Finally, out of the remaining 375 datasets, 20 were excluded, i.e., about 3%, which correspond to those that had less than 70% overall accuracy on the questions. This resulted in a total number of 355 participants in the included data set, from which 123 male, 166 female, 66 declined to provide demographic information; mean age was 41.38 (*SD* = 12.92).

This study was carried out in accordance with the recommendations of the NYU University's Institutional Review Board (IRB). All subjects gave written informed consent before beginning the experiment in accordance with the Declaration of Helsinki.

### Materials

The experimental stimuli consisted of a total of 144 sentences, divided into the 6 different types of structures exemplified in Table 1 (6 types × 24 sentences = 144).

We have been exhaustive in including as many conditions as structural differences there are between the two models. Thus, sentence types were selected on the basis of the basic syntactic structures attributed to them in the two models under study. Structuring them into 6 types covers all (in)transitive and small clause patterns. For instance, even though creation verbs (C3) and strong transitives (C5) surface as transitives, they have the same structure in the *interpretive* model, but they are attributed different syntactic structures in the *generative* approach, already so since Hale and Keyser's (1993) seminal work. Therefore, the two models predict different priming effects between these conditions as well as in their interaction with the rest of conditions. To wit, as shown in Table 2, whereas creation verbs (C3) and strong transitives (C5) are predicted to prime each other in the *interpretive* model, they are not in the *generative* model. Likewise, although creation verbs (C3) would prime unergatives (C1) in the *generative* framework, strong transitives (5) do not prime them; neither creation verbs (C3) nor strong transitives (C5) would prime unergatives in the *interpretive* theory.

Specific verbs were selected on the basis of the frequency rates of the syntactic patterns they may appear in as reported in the VALEX subcategorization corpus (Korhonen et al., 2006). Specifically, unergative verbs (C1/C2) were chosen on the basis of their low frame frequency with NP complements (frequency lower than 0.15). Creation verbs, Location/Locatum predicates^6^, and *With*-Small clause structures were selected from among those with the highest frame frequency rate in the corresponding structure. Strong transitives were chosen on the basis of their high frame frequency with NP complements (frequency higher than 0.83). In addition, combinations of V+N and A+N were checked against the Corpus of Contemporary American English's (COCA) lexical collocations (Davies, 2008). We also took the definiteness of the NP in Segment 3 into account, as it has been shown that it plays a role in language processing (e.g., Warren and Gibson, 2002). All sentences were further tested against native speaker judgments to confirm naturalness.

We designed a structural priming experiment with six different conditions on sentence structure to run a self-paced reading language comprehension study over Mechanical Turk. Structural priming was tested within and across sentence types using a priming paradigm where each target item also served as a prime sentence for the next target item. In addition, we included an attention task and control condition, which was organized as follows. Every set of 24 sentences had 6 sentences linked to a two-choice comprehension question of the type in (23–24), with a total of 36 questions. These questions served the double function of being an attention task and a control condition to obtain additional reading times from the same prime/target sentences in non-primed contexts.

(23) He dodged a corporate tax in the UK.
(24) Did he evade the tax or pay it?
  1. evade it
  2. pay it

A complete list of 144 sentences by condition with the corresponding 36 attention tasks linked to 6 individual sentences on each condition is provided as Supplementary Material.

### Procedure–study implementation

Sentences of each condition (24 × 6 = 144) were separated into 4 segments, Segment 1-Segment 4: Subject (Segment 1), Verb (Segment 2), First Complement (Segment 3), Second Complement (Segment 4).

**Table:** 

	**Conditions**		**Segment 1**	**Segment 2**	**Segment 3**	**Segment 4**
			**NP**	**V**	**NP(/PP)**	**PP**
(25)	**C1**.	Unergative	The dog	barked	in a quiet park	at night.
(26)	**C2**.	Cognate	The man	dozed	a restful doze	on the train.
(27)	**C3**.	Creation	The cook	baked	a carrot cake	with spelt flour.
(28)	**C4**.	Location/Locatum	The girl	saddled	a wild horse	in the farm.
(29)	**C5**.	Strong transitives	The athlete	ignored	a slight niggle	in his knee.
(30)	**C6**.	*With*-Small clause	The worker	loaded	a rail wagon	with hay.

We used a running priming paradigm, so that each target sentence served as the prime sentence for the next target item (e.g., Segaert et al., 2012, 2013). Sentences were organized in 3 blocks of trials, with 6 block orderings. Trials were randomized within blocks, so that the conditions followed each other in a random order that was different for each participant. One in every 6 trials was followed by a two-choice comprehension question.

The study was created in Ibex Farm. Participants were shown instructions and they completed a short practice round before the actual experiment started. As a self-paced reading experiment, participants determined the rate at which sentential segments were presented on the monitor by pressing a button, which allowed us to measure reading times at each segment. Each segment was presented sequentially in the center of the screen with 400 ms between each sentence.

### Preprocessing and statistical model analyses

Before running the statistical analysis, we calculated the average reading time for each participant, for each segment and then we removed outliers based on this. That is, we excluded trials with reaction times greater than 2 standard deviations from the participant's respective mean. We also excluded the first trial of each block, because it did not fit into either our primed conditions or the unprimed question conditions.

Based on our basic hypothesis that differences in priming effects are expected at the point where both models differ structurally, we decided to focus on the reading times of Segment 3, the first constituent after the verb. Depending on the model, in that position we have a DP complement, a small clause complement or an adverbial. The validity of this hypothesis seemed to be confirmed by the results from a preliminary Analysis of Variance (ANOVA) (6 × 6 within subjects; Factors: condition + previous_condition) and visual inspection of the plots, as these differences between sentence types seemed most pronounced in this segment. The controlled analyses that follow all have Segment 3 reading time as the outcome/response variable.

The main analysis of the data was conducted through two different forms of Analyses of Covariance (ANCOVA), for single priming trials and for double priming trials, respectively, and a linear mixed effects regression model, to tailor the two different theories. The use of statistical control allowed us to measure different variables in addition to the independent variables of interest and to control for unexplained variation. For instance, the ANCOVA allows us to have factors as predictors rather than just continuous variables as in a linear regression model.

In both the ANCOVA and the Linear mixed effects regression analysis, the null hypothesis is that all coefficients equal 0. That means that none of the independent variables have any relationship with or effect on the dependent variable, i.e., on the reading time of Segment 3. The alternative hypotheses are that at least one independent variable is predictive of the dependent variable; thus, at least one coefficient does not equal 0.

The statistical analysis was performed using the R Core Team (2015) software program with packages lme4 and lmerTest.

#### Single priming analysis: ANCOVA 1.0

Following standard procedures, in order to control or minimize the effects of extraneous sources of variance, we included the nuisance variables listed in (31) as covariates. Note that trial order was included as it may account for some of the variance in reading time, e.g., participants may get faster as they proceed or they may change their strategy later in the experiment. Random intercepts by subject and by item were also included in the models.

(31)
  - trial order
  - verb frequency
  - reading times (RT) of previous segment
  - RT of same segment in previous trial

We coded two variables, V_I_-V_G_, on the basis of how each theory, interpretive and generative, groups the various conditions, (32).

(32)
  - V_I_–Sentence Types: Unergatives (C1), DP/Root (C2–C5), Small Clause (C6).
  - V_G_–Sentence Types: DP/Root (C1–C3), Small Clause (C4–C6).

That means that we took the 6 initial conditions, C1-C6, and grouped them according to the syntactic patterns attributed to them in each model. This results in a three-level classification of our six conditions for the interpretive model and two levels for the generative approach.

The two variables were included as predictors in an ANCOVA model, with log-transformed frequency, trial order, previous trial RT, and previous segment (of the same trial) RT as controls/covariates.

To control for type 1 error rate, we used nested models in log-likelihood ratio tests in order to determine the contributions of individual variables, a standard method for dealing with type 1 error in multiple regression models.

#### Double priming analysis: ANCOVA 2.0

As pointed out in Tooley and Traxler (2010), priming effects without lexical repetition in comprehension were reported in the context of double primed sentences in Thothathiri and Snedeker's (2008a) eye-tracking experiments with ambiguous double object and dative constructions. Thus, we designed a second ANCOVA model in order to test whether structural priming with unambiguous active sentences might be aided or affected in trials where two previous primes of the same category precede the target trial.

Including the same variables as in the previous ANCOVA 1.0 model in (31)–(32), we constructed a new ANCOVA model 2.0 by adding the two new variables in (33) and their interaction with the variables associated with their respective models (V_I_, V_G_) in (32). For each new variable, trials were coded as follows:

(33)
  - If the trial was preceded by TWO trials of the same condition (same as each other, not as the current trial, according to the interpretive theory), then the trial was coded as the condition of those 2 preceding trials (e.g., “Preceded by 2 Unergatives”). Otherwise, the trial was coded as “N/A.”
  - If the trial was preceded by TWO trials of the same condition (same as each other, not as the current trial, according to the generative theory), then the trial was coded as the condition of those 2 preceding trials (e.g.,“Preceded by 2 DP/Root”). Otherwise, the trial was coded as “N/A.”

#### Model-tailoring analysis: linear mixed effects regression model

One of the potential limitations of our ANCOVAs quantitative analyses has to do with the fact that the dependent variable in the generative model had fewer levels than in the interpretive model of Marantz, which could perhaps inherently restrict its ability to capture variance. To avoid this, we designed a linear mixed effects regression model that would test for syntactic priming on the basis of the grouping of conditions in each model. We took the same control variables as in our previous ANCOVA analyses, and coded two additional binary variables for each model, as in (34).

(34)
  - V_I_–Binary Priming (coded as 1 for primed, 0 for unprimed)
  - V_G_–Binary Priming (same coding scheme)

Based on the predictions of each model in terms of priming relations between conditions as depicted in Table 2, we coded the variables in (34) as the two primary variables in (35).

(35)
  - Primed: Anything that has a check mark in Table 2 was coded as 1
  - Unprimed: Anything that has a cross mark in Table 2 was coded as 0

We coded two other binary variables for each model, V_I(G)_– Same Previous and V_I(G)_ Same Two Previous [see (30a) and (30b) respectively]. Note that the subscript I(G) indicates that there were two corresponding variables calculated, one based on each model.

(36)
  - V_I(G)_–Same Previous = binary variable coded 1 for trials where the previous trial was the same condition as the current, based on the respective model; 0 if not
  - V_I(G)_–Same Two Previous = binary variable coded 1 for trials where the previous two trials were the same condition as the current, based on the respective model; 0 if not

## Results

Table 4 shows the log-transformed mean response time and standard deviation for all individual conditions in all conditions of individual priming by condition (6 target × 6 previous).

**Table 4:** Log-transformed mean reading time (St. Dev.): Condition × Previous Condition.

	**PREVIOUS CONDITION**							
		**C1**	**C2**	**C3**	**C4**	**C5**	**C6**	**Average**
Condition	C1	6.119 (0.636)	6.098 (0.616)	6.096 (0.628)	6.09 (0.645)	6.093 (0.624)	6.084 (0.594)	6.097
	C2	6.096 (0.588)	6.091 (0.626)	6.069 (0.573)	6.113 (0.602)	6.084 (0.622)	6.092 (0.649)	6.091
	C3	6.054 (0.558)	6.036 (0.608)	6.057 (0.575)	6.054 (0.594)	6.052 (0.580)	6.048 (0.605)	6.050
	C4	6.049 (0.609)	6.053 (0.574)	6.054 (0.613)	6.071 (0.572)	6.053 (0.637)	6.054 (0.537)	6.056
	C5	6.074 (0.603)	6.066 (0.609)	6.064 (0.614)	6.068 (0.598)	6.093 (0.578)	6.086 (0.597)	6.075
	C6	6.071 (0.608)	6.076 (0.590)	6.067 (0.612)	6.065 (0.593)	6.068 (0.575)	6.085 (0.578)	6.072
Average		6.077	6.070	6.068	6.077	6.074	6.075	

**Figure 1:**
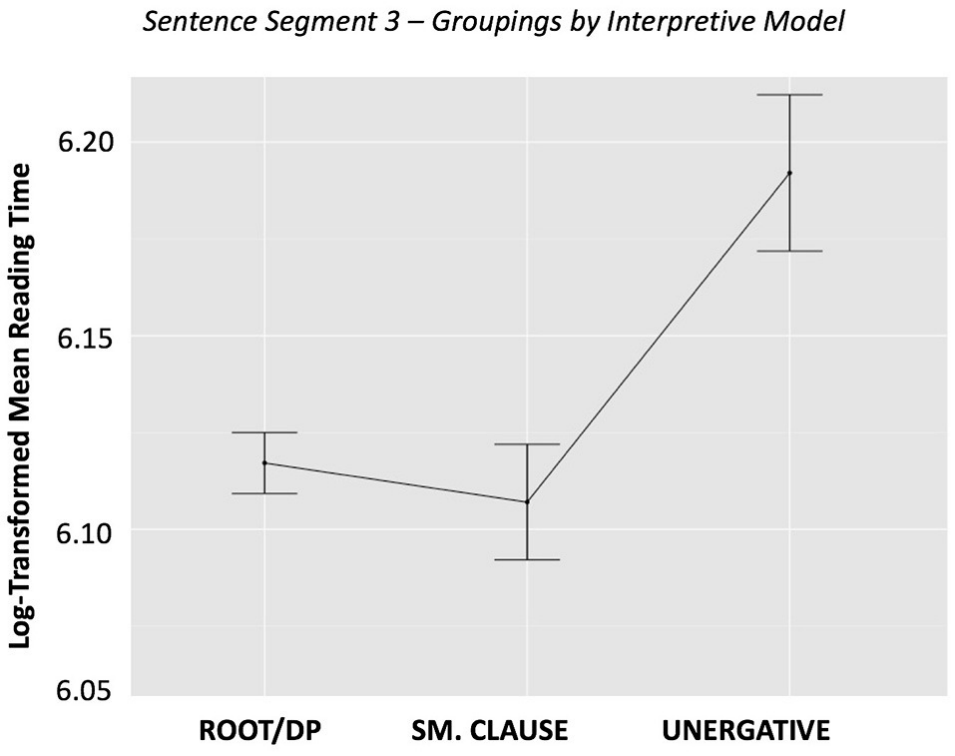
Sentence Segment 3–Groupings by *Interpretive* Model, by condition. This figure shows mean reading times for sentence grouping categories based on the *interpretive* model. Error bars represent the standard error of the mean.

**Figure 2:**
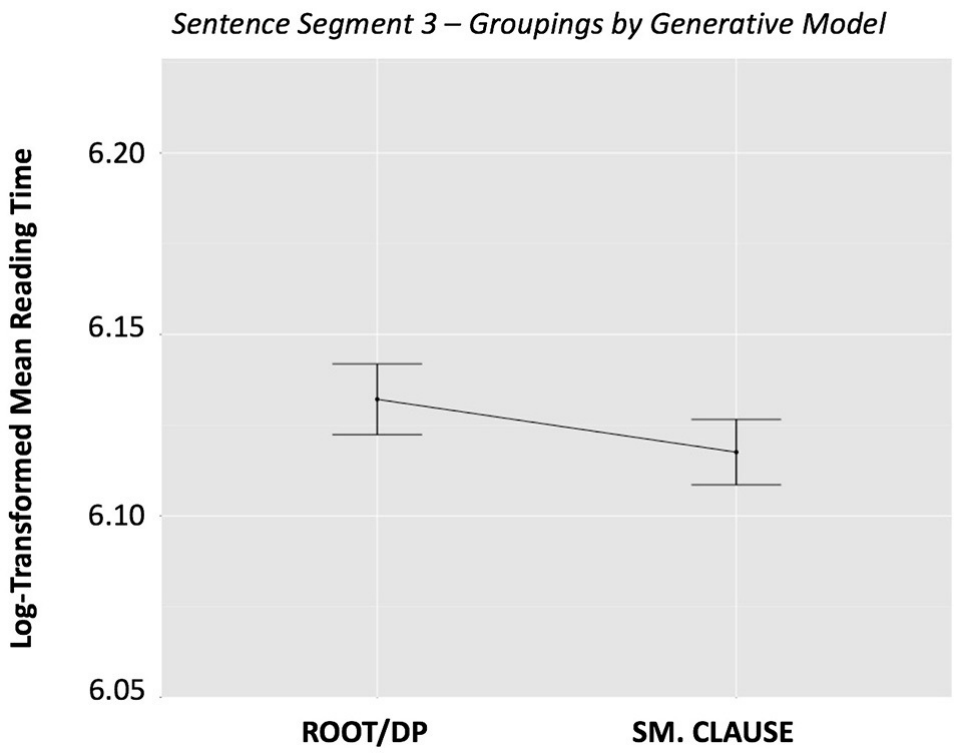
Sentence Segment 3–Groupings by *Generative* Model, by condition. This figure shows mean reading times for sentence grouping categories based on the *generative* model. Error bars represent the standard error of the mean.

The single priming analysis, ANCOVA 1.0, revealed that categorization based on the interpretive model (V_I_ – Sentence Type) was a significant predictor of reading time in Segment 3 (*p* = 0.012). In contrast, categorization based on the generative model (V_G_–Sentence Type) did not significantly affect reading time (*p* = 0.1379). The raw reading times (i.e., not taking into account random effects or nuisance variables) are graphed in Figures 1, 2. The graphs should be interpreted cautiously, as they do not reflect the influence of random intercepts or nuisance variables, which were included in the ANCOVA, and thus are subject to potential confounds.

In the double priming analysis, ANCOVA 2.0, a “full” statistical model, i.e., one including the interaction between sentence type and previous (x2) sentence type, was tested against a model excluding the respective interaction terms, for both the generative and the interpretive theories. This initially gave us a null result. So, the contribution of the interpretive model interaction was not significant (*p* = 0.649), nor was the contribution of the generative model interaction (*p* = 0.863).

**Figure 3:**
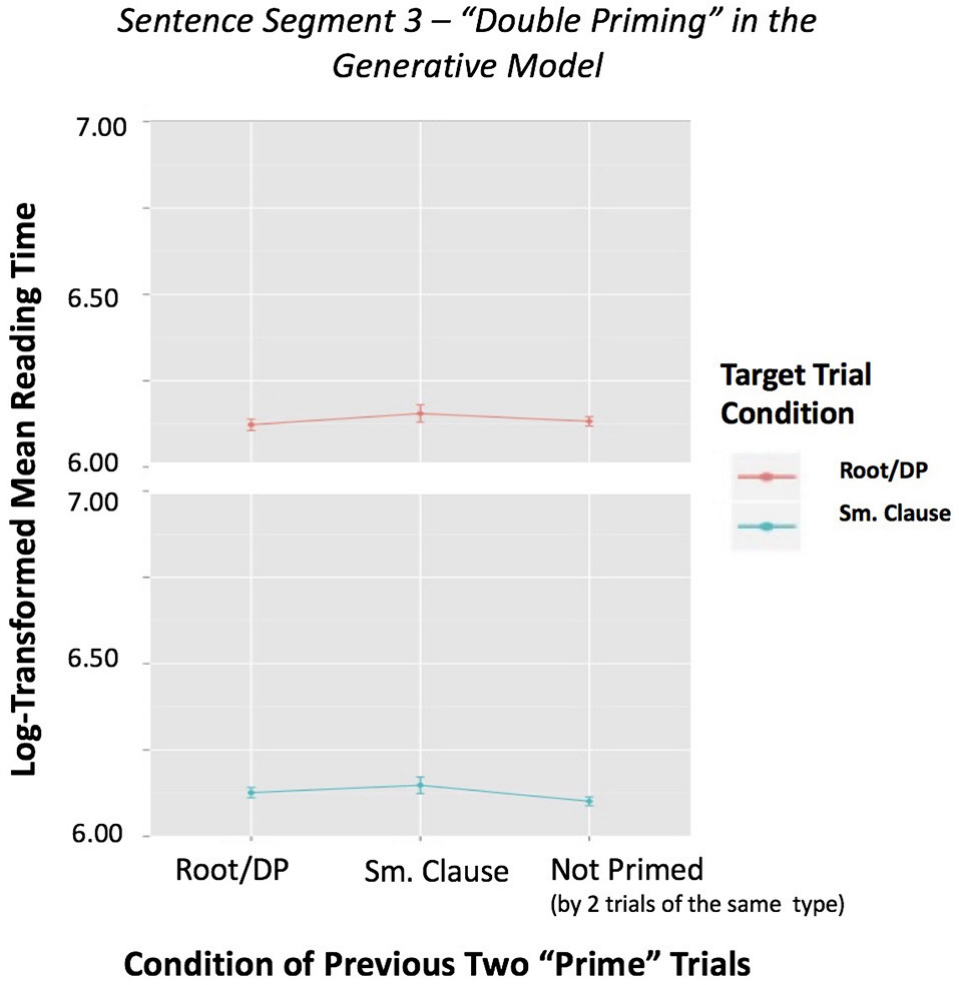
Sentence Segment 3–“Double Priming” in the *Generative* Model by Two Previous Conditions, with reading time of Segment 3 as dependent variable. This figure shows mean reading times for each sentence category preceded by two trials of the same condition based on the *generative* model. It also includes the reading times of Segment 3 when not preceded by two trials of the same type. Error bars represent the standard error of the mean.

**Figure 4:**
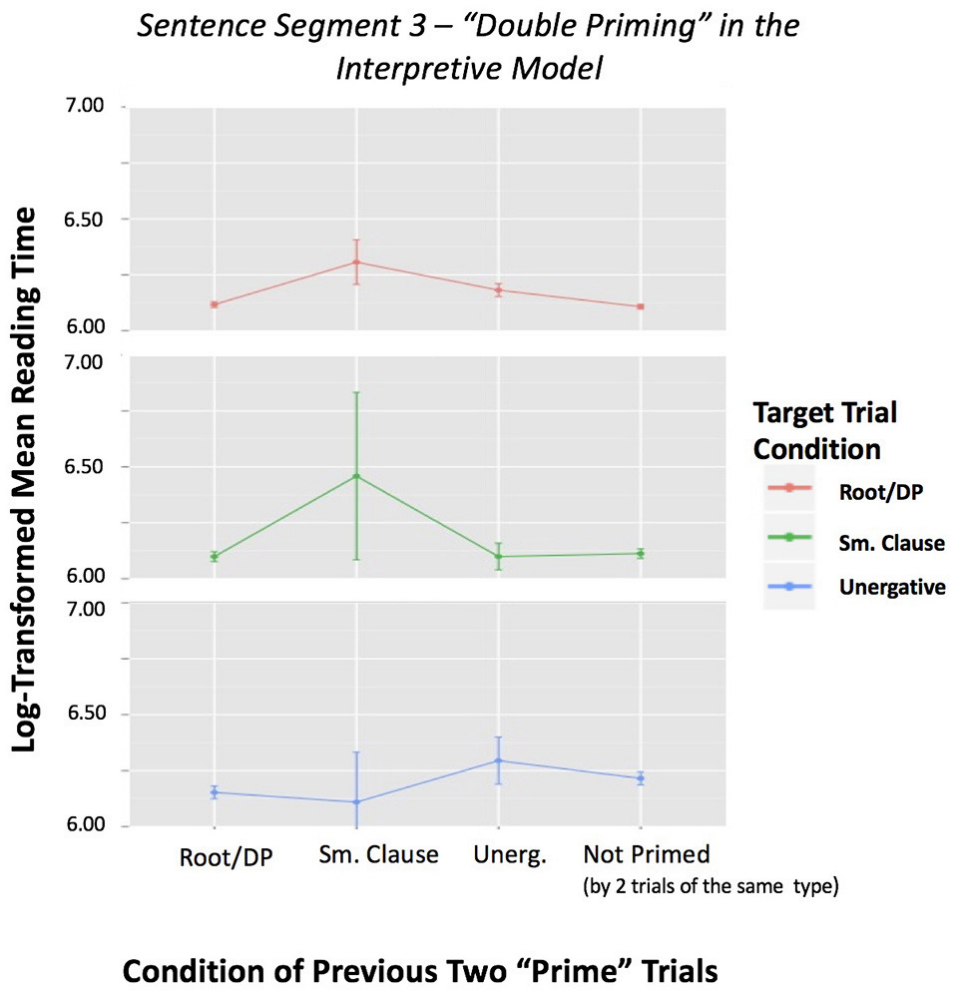
Sentence Segment 3–“Double Priming” in the *Interpretive* Model by Two Previous Conditions, with reading time of Segment 3 as dependent variable. This figure shows mean reading times for each sentence category preceded by two trials of the same condition based on the *interpretive* model. It also includes the reading times of Segment 3 when not preceded by two trials of the same type. Error bars represent the standard error of the mean.

However, when we removed the random effects structure, keeping trial order as a covariate, we obtained again significant effects. The contribution of the *interpretive* model interaction was significant (*p* = 0.0037), whereas the contribution of the *generative* model interaction was not significant (*p* = 0.756). Even though these results should be interpreted with caution due to the simplified status of the model, they tentatively show a stronger predictive power of the *interpretive* approach. Figures 3, 4 depict the interaction of sentence type by previous sentence type, according to each of the two models, with reading time of Segment 3 as the dependent variable.

As for our last statistical analysis, our model-tailoring analysis, no statistically significant effects were found in the linear mixed effects regression model, regardless of whether the random effects structure is included in the model, as shown in (37–38).

(37) Without considering random effects
  - *Interpretive* model (*p* = 0.1078)
  - *Generative* model (*p* = 0.2999)

(38) With random effects
  - *Interpretive* model (*p* = 0.1766)
  - *Generative* model (*p* = 0.565)

## Discussion

Our first ANCOVA 1.0 analysis on single priming effects revealed a distinction between the two models. As shown in the relevant plots, there is no significant separation between conditions for the *generative* model, but we do observe separation in the *interpretive* model, particularly for the unergatives. In that sense, this effect of the *interpretive* model may be primarily driven by the fact that, in this approach, unergatives are considered to be their own category, whereas they are integrated in one of the groupings in the *generative* model, together with cognate object structures and verbs of creation.

Although the initial ANCOVA 2.0 analysis on double priming revealed no significant effects, after removing the random effects we observe a stronger predictive power of the interpretive approach. Figure 3 shows no evidence that some set of V NP PP structures, those grouped under location/locatum sentences (C4) and strong transitives (C5), behaves like a small clause (SC) or that unergatives (C1) look like transitives (C2-C3) in the *generative* model. However, in Figure 4, we can observe effects for the small clause condition for the *interpretive* model. That is, two small clause sentences (C6) before a small clause sentence (C6) causes a slow-down in the reading times of Segment 3, while two standard V NP PP sentences (C2-C3-C4-C5) before a small clause type sentence (C6) causes a significant speed up in Segment 3 reading times^7^.

Even though results were not significant, nor even trending, the linear mixed effects regression model is likely our most reliable model, because we have reduced the number of levels for the variables we are testing to just two for both models. It is worth noting that, as shown in (37) and (38), the effect size for the interpretive model is consistently larger than that of the generative approach, and the *p*-values of the interpretive model are consistently smaller, regardless of whether the random effects structure is included in the model.

We should note that even though the linear mixed effects regression model is most likely a more unbiased analysis, it does not allow us to investigate differences in priming between conditions, which is what we did in our ANCOVA 1.0, nor does it allow us to look at the interaction between the trial type and the prime type, as we showed in Figures 3, 4, resulting from our ANCOVA 2.0. Thus, the different statistical models we have employed do not exclude each other, but rather complement each other's limitations and they all seem to point toward a stronger predictive power of the *interpretive* approach.

## Conclusions

We have employed the experimental method to assess two competing linguistic accounts of the syntactic representation of the argument structure of (in)transitive structures on the basis of their divergent predictions with respect to sentence processing under conditions of syntactic priming. The design of our experiment makes use of on-line behavioral methods like self-paced reading, experimental techniques like priming, quantitative tools like frequency-based corpora, and sophisticated statistical control typical of experimental research in cognitive science to obtain reading time measures that allow us to effectively characterize theories about the representation and processing of syntactic phenomena. We have obtained significant results that point to a stronger predictive power of Marantz's interpretive theory over Acedo-Matellán and Mateu's generative model. Likewise, we have found no evidence in favor of the main claims of the generative analysis that some set of V NP PP structures behave like the small clauses or that unergatives are underlying transitives.

We have made a novel use of structural priming as a tool to discriminate among linguistic theories. A second novelty of the experiment lies in the structures we focus on, i.e., the empirical domain of the study. Whereas the central empirical issue in structural priming studies has mostly been how ambiguities arise and are resolved in incrementally disambiguating sentence processing, our empirical focus is on processing basic active simple (in)transitive structures. One of our main findings is thus that there is structural priming in comprehension between basic structures without lexical boost.

To conclude, our controlled behavioral experimentation supports quantitative approaches to the study of I-language that advocate for the complementarity of psycholinguistic and theoretical methodologies to help us determine the nature of linguistic phenomena.

## Limitations and further research

As already mentioned above, one of the potential limitations of the model variables relates to the number of levels, three in Marantz's *interpretive* model vs. two in Acedo-Matellán and Mateu's *generative* model in the ANCOVAs, which could inherently restrict their ability to capture variance and as a consequence have an effect of grouping conditions by theory on our findings. Note, however, that the analysis where we test the ungrouped condition variable, i.e., the variable coded as 1–6, with six levels, is likewise not significant (*p* = 0.11). Thus, it does not seem that adding more levels to the categorical predictor would improve the analysis. Yet, we should still interpret these results cautiously.

More data may be needed to see separation between the other conditions in the interpretive model or in the generative model, but that will likely be a focus of the future of this project. With respect to our ANCOVA 2.0, we only had few trials preceded by 2 trials of the same condition as the current trial, therefore, more data must be gathered to obtain reliable results in this direction. At this point, while we have preliminary effects showing the interpretive model is a better predictor, this appears to be only based on one aspect of the model, and we may not currently have enough statistical power to look at all aspects of the model.

We have also detected an unexpected slow-down in response times for primed trials that must be further investigated.

## Author contributions

IO and AM designed the research; IO drafted the work; all authors critically revised the drafts; VS and AM designed the MTurk; VS ran the MTurk and the statistical models on R; VS pre-processed the data. All authors analyzed the data, interpreted the results and wrote the final paper.

### Conflict of interest statement

The authors declare that the research was conducted in the absence of any commercial or financial relationships that could be construed as a potential conflict of interest.

## Footnotes

^1^Our use of the labels *interpretive* and *generative semantics* should be strictly understood in the sense just explained in the main text, and not as the two approaches to semantics of the 70 s within the theory of transformational grammar, e.g., Katz (1971).

^2^The possibility of having priming of lexical argument structure of the type proposed in Trueswell and Kim (1998), rather than syntactic priming, is thus excluded in these models.

^3^Other theories of argument structure, such as those within monostratal theories of syntax like Generalized Phrase Structure Grammar or Role and Reference Grammar, do not share the same basic assumptions with respect to syntactic argument structure building, and could not be easily integrated within our experimental study.

^4^In Hale and Keyser (1993, 1998, 2002), syntactic configurations corresponded to pre-syntactic abstract structures, i.e., generated at *l(exical)*-syntax, prior to *s(yntactic)*-syntax. However, they are analyzed as surface structures generated in syntax proper in Acedo-Matellán (2010) and Acedo-Matellán (2010), Acedo-Matellán and Mateu (2013), as explicitly stated in e.g., Acedo-Matellán (2010, p. 52).

^5^That means that either (i) some subjects completed HITs without doing the experiment, or (ii) some of the datasets did not get saved on the Ibex server.

^6^Location and Locatum verbs were first selected from among Clark and Clark's (1979, pp. 769–773) classification. There are 12 sentences with Location verbs and 12 with Locatum verbs.

^7^It should be noted that our graphs do not technically include random effects and order, nor any of our other control variables; to the best of our knowledge, there is no way to cleanly integrate them into a plot. This is why the graphs may not look particularly informative.
