# Supplementary material for: Syntactic Priming As a Test of Argument Structure: A Self-paced Reading Experiment
Source: Front Psychol. 2017 Aug 15;8:1311. doi: 10.3389/fpsyg.2017.01311 (PMC5559723; doi:10.3389/fpsyg.2017.01311)
Supplement: Supplementary file 1 [file DataSheet1.PDF]

			UNERGATIVES - NO OBJECT				QUESTIONS
C1	1		The dog	barked	in a quiet park	at night.	
C1	2	*	The machine	bleeped	in an empty office	at noon.	Was it a light or a sound?
C1	3		The nestlings	cheeped	in a public garden	at night.	
C1	4		The bird	chirped	in a rose bush	at daybreak.	
C1	5	*	The man	chortled	in a long queue	with glee.	Was he thrilled or sad?
C1	6		The frog	croaked	in a thick fog	at night.	
C1	7		The woman	cried	like a little baby	at night.	
C1	8		The man	died	at a foster home	without pain.	
C1	9	*	The man	dozed	on a wide porch	in the sunshine.	Was the man napping or sitting in the sun?
C1	10		The teacher	frowned	in a funny way	with annoyance.	
C1	11	*	The speaker	giggled	with great delight	at the symposium.	Was she laughing or talking?
C1	12		The patient	groaned	in stunned silence	under the pressure.	
C1	13		The woman	guffawed	with glee	on the phone.	
C1	14		The baby	gurgled	with pure joy	in his crib.	
C1	15		The girl	hiccoughed	in a language class	with laughter.	
C1	16	*	The artist	huffed	with sudden impatience	on the microphone.	Was he singing or breathing loudly?
C1	17		The dean	laughed	in a rude fashion	at the meeting.	
C1	18		The couple	slept	on an old couch	at night.	
C1	19		The car	sputtered	at high speeds	like a used moped.	
C1	20		The twins	tittered	in perfect unison	during the screening.	
C1	21	*	The device	whirred	at a high speed	in the background.	Was it a noise or a flashlight?
C1	22		The child	yapped	with a good buddy	over the weekend.	
C1	23		The audience	yawned	in obvious boredom	during the talk.	
C1	24		The cat	yowled	in mock alarm	around the fence.	

* = linked to task

			UNERGATIVES + COGNATE OBJECT				QUESTIONS
C2	1		The dog	barked	a warning bark	in the garden.	
C2	2		The engine	bleeped	an annoying bleep	at night.	
C2	3		The insect	cheeped	a happy cheep	at daybreak.	
C2	4	*	The robin	chirped	a noisy chirp	in the morning.	Was it loud or quiet?
C2	5		The farmer	chortled	a hearty chortle	in the backyard.	
C2	6		The frog	croaked	an ugly croak	in the garden pond.	
C2	7		The spectator	cried	a desperate cry	during the movie.	
C2	8		The priest	died	a peaceful death	at home.	
C2	9		The man	dozed	a restful doze	on the train.	
C2	10		The doctor	frowned	a severe frown	in front of him.	
C2	11		The professor	giggled	a timid giggle	at the conference.	
C2	12		The child	groaned	a painful groan	in protest.	
C2	13	*	The receptionist	guffawed	a loud guffaw	on the phone.	Was she cheerful or unhappy?
C2	14		The baby	gurgled	a rumbling gurgle	in his crib.	
C2	15		The girl	hiccoughed	a violent hiccough	during lunch.	
C2	16	*	The singer	huffed	an audible huff	on the microphone.	Was he in a bad or in a good mood?
C2	17		The instructor	laughed	an infectious laugh	at the workshop.	
C2	18		The couple	slept	a deep sleep	at the motel.	
C2	19		The motor	sputtered	a soft sputter	at high speeds.	
C2	20	*	The candidate	tittered	an inappropriate titter	in the interview.	Was he chuckling or shouting?
C2	21		The gadget	whirred	a constant whirr	in the background.	
C2	22	*	The student	yapped	an endless yap	with his buddies.	Was he gossiping or eating a lot?
C2	23		The artist	yawned	a big yawn	during the show.	
C2	24	*	The boy	yowled	an angry yowl	in response.	Was he writing or screaming?

			VERBS OF CREATION				QUESTION
C3	1		The trainer	assembled	a winning team	with our help.	
C3	2		The cook	baked	a carrot cake	wih spelt flour.	
C3	3		The architect	built	a detached house	in the countryside.	
C3	4	*	The girl	carved	a wooden toy	at school.	Did she find it or chisel it?
C3	5		The writer	coined	a novel phrase	in her book.	
C3	6	*	The secretary	compiled	a comprehensive report	for the company	Did he gather or distribute the information?
C3	7	*	The musician	composed	a choral symphony	in a week.	Did she write it or correct it?
C3	8		The learner	constructed	a long sentence	in Chinese.	
C3	9		The housemaid	cooked	a tasty meal	for the whole family.	
C3	10		The director	created	a strategic plan	with a partner.	
C3	11		The woman	designed	an interactive website	for a company.	
C3	12		The child	drew	a big circle	on her notebook.	
C3	13		The man	formed	a political party	in the town.	
C3	14		The pupils	imagined	a fairer world	for everyone.	
C3	15		The nun	invented	a different story	for her brother.	
C3	16	*	The man	knitted	a winter sweater	in the basement.	Did he make it or wash it?
C3	17		The manager	made	a common mistake	in her calculations.	
C3	18		The woman	painted	a large mural	in the alley.	
C3	19		The company	produced	sewing machines	in Germany.	
C3	20	*	The artist	sculpted	a human figure	for the museum.	Did she carry it or model it?
C3	21		The engineer	sketched	an original idea	for a research project.	
C3	22		The student	typed	a formal letter	for the language class.	
C3	23	*	The tailors	wove	a unique pattern	with organic cotton.	Did they develop it or identify it?
C3	24		The researcher	wrote	a joint paper	with her husband.	

			**LOCATION/LOCATUM VERBS (*saddle/shelve*)**				**QUESTION**
C4	1	*	The army	dammed	a flooded river	in Austin.	Did they block it up or crossed it?
C4	2		The bird	feathered	a cozy nest	on a tree.	
C4	3		The government	forested	a remote mountain	in Africa.	
C4	4		The carpenter	framed	a great picture	at home.	
C4	5		The chef	greased	a cooking pan	on all sides.	
C4	6	*	The farmer	harnessed	a small donkey	in the stall.	Did she hitch it (up) or ride it?
C4	7		The company	labeled	a specific product	at the back.	
C4	8	*	The worker	patched	a light jacket	by hand.	Was he sewing or ironing?
C4	9		The factory	poisoned	a giant rat	in the basement.	
C4	10		The builder	roofed	a wooden house	in the summer.	
C4	11		The girl	saddled	a wild horse	in the farm.	
C4	12		The man	salted	a fresh salad	in the kitchen.	
C4	13	*	The pilot	bagged	a real bargain	in the shop.	Did he purchase it or just saw it?
C4	14		The family	bottled	red wine	in the vineyard.	
C4	15		The firemen	caged	a young tiger	in the zoo.	
C4	16		The secretary	charted	a scenic route	at work.	
C4	17		The children	corralled	a stubborn goat	in the farm	
C4	18		The clerk	filed	a global report	at the court.	
C4	19		The police	jailed	a political prisoner	in that country.	
C4	20		The boy	pocketed	a cell phone	at the store.	
C4	21		The student	shelved	a recent book	in the library.	
C4	22		The hiker	shouldered	a heavy knapsack	without difficulty.	
C4	23	*	The woman	staged	a solid play	in Paris.	Is she a viewer or an artist?
C4	24	*	The player	strung	a tennis racket	during the break.	Did he break it or repair it?

12 Locatum + 12 Location

			STRONG TRANSITIVE VERBS				QUESTION
C5	1	*	The president	appeased	a pressure group	in her speech.	Did she upset them or calm them down?
C5	2		The engineer	automated	a complex process	in the factory.	
C5	3	*	The doctor	curbed	a growing epidemic	in the country.	Did he release it or contain it?
C5	4		The administrator	defied	an European law	in her proposal.	
C5	5	*	The businessman	dodged	a corporate tax	in the UK.	Did he evade the tax or pay it?
C5	6		The director	embraced	a new proposal	without hesitation.	
C5	7	*	The priest	extolled	a cardinal virtue	in the letter.	Did they criticize it or praise it?
C5	8		The assistant	faced	a huge problem	in the office.	
C5	9		The manager	guaranteed	a net profit	at the end of the term.	
C5	10		The athlete	ignored	a slight niggle	in his knee.	
C5	11		The girl	mimicked	a husky voice	on a phone call.	
C5	12		The company	mortgaged	a rural farm	in the summer.	
C5	13		The boss	offered	a good job	in the company.	
C5	14		The government	preserved	a unique species	in the area.	
C5	15		The student	raised	a shaky hand	over her eyes.	
C5	16	*	The expert	rebutted	a false point	in a debate.	Did he refute it or support it?
C5	17		The customer	rescinded	a valid contract	after the trial.	
C5	18		The scientist	sectioned	a rare specimen	in the lab.	
C5	19		The shoemaker	soled	a flat shoe	on the spot.	
C5	20		The woman	took	an old radio	at the shop.	
C5	21		The economy	triggered	a global crisis	in Europe.	
C5	22		The salesman	vended	salt fish	in the stalls.	
C5	23		The designers	violated	a basic rule	on their blog.	
C5	24	*	The boat	weathered	a sudden storm	on the coast.	Did it sink or come through?

			SPRAY/LOAD *with*-ALTERNATION				QUESTIONS
C6	1		The cook	brushed	a grill rack	with oil.	
C6	2	*	The guest	crammed	a huge ashtray	with cigarette butts.	Was it overflowing or almost empty?
C6	3		The corporation	crowded	a natural shoreline	with buildings.	
C6	4	*	The artist	dabbed	a giant mural	with synthetic paint.	Did he spray it or pat it?
C6	5		The girl	daubed	a white wall	with paint.	
C6	6		The decorator	draped	a small table	with fine fabric.	
C6	7		The plumber	filled	a separate container	with cold water.	
C6	8	*	The waiter	heaped	an empty plate	with boiled rice.	Was it meager or abundant?
C6	9		The biologist	infected	a tropical plant	with a microorganism.	
C6	10		The doctor	injected	a large pimple	with cortisone.	
C6	11	*	The council	jammed	a local school	with new students.	Was it overcrowded or almost vacant?
C6	12		The worker	loaded	a rail wagon	with hay.	
C6	13		The researcher	pumped	a healthy body	with steroids.	
C6	14	*	The man	rubbed	a baking dish	with garlic	Did he cut the garlic or smear it?
C6	15		The farmer	seeded	a hunting plot	with an annual mixture.	
C6	16		The housemaid	soaked	a clean cloth	with gasoline.	
C6	17		The child	spattered	a new dress	with mud.	
C6	18		The woman	sprayed	a cookie sheet	with vegetable oil.	
C6	19		The tourist	spread	a hot biscuit	with raspberry jam.	
C6	20		The chef	sprinkled	a cutting board	with flour.	
C6	21		The nurse	squirted	a cotton swab	with redness-reducing eyedrops.	
C6	22		The firm	stocked	a writing centre	with blank books and crayons.	
C6	23	*	The camper	stuffed	a zipper-lock bag	with clothes	Was it full or half empty?
C6	24		The florist	wrapped	a straw basket	with cellophane.	
